# Prediction models for stunting at 2-years-old from Indonesian newborn population

**DOI:** 10.1186/s12887-025-06096-4

**Published:** 2025-10-02

**Authors:** Devi Azriani, Dwi Agustian, Yenni Zuhairini, Intan Nurma Yulita, Meita Dhamayanti

**Affiliations:** 1 Doctoral Student, Faculty of Medicine, Universitas Padjadjaran, Bandung, Indonesia; 2 Health Polytechnic of the Ministry of Health, Jakarta 1, Jakarta, Indonesia; 3 Department of Public Health, Faculty of Medicine, Universitas Padjadjaran, Bandung, Indonesia; 4 Faculty of Mathematics and Natural Sciences, Universitas Padjadjaran, Bandung, Indonesia; 5 Department of Child Health, Faculty of Medicine, Universitas Padjadjaran, Bandung, Indonesia

**Keywords:** Indonesia, Models, Newborns, Prediction, Stunting

## Abstract

**Background:**

Stunting in children is a health problem, especially in developing countries, such as Indonesia. The lack of information-based early preventive measures resulted in an insignificant reduction in stunting. This study aimed to develop a prediction model for stunting at 2-years old in an Indonesian newborn population.

**Method:**

Various machine learning algorithms as the core of artificial intelligence technology, under the Cross-Industry Standard Process for Data Mining (CRISP-DM) conceptual framework, were used to build a prediction model using data of 5093 children with 23 predictor variables from the Indonesian Family Life Survey (IFLS) open database. Model prediction performance was evaluated using F1 scores, area under the receiver operating characteristic curve (AUC), sensitivity, and accuracy. Confusion matrices were used to calculate the positive and negative predictive values and evaluate the implications of the final prediction model in public health and clinical practice. Explanatory-risk factor model was developed using multivariable logistic regression.

**Result:**

The best model to predict stunting statistically, which contains the six best predictor variables, is k-nearest neighbor (kNN) with an F1 value of 84.5%, compared to random forest, neural network, decision tree, and naïve Bayes with F1 values of 80.5%, 71.2%, 68.7%, and 65.8%, respectively. The positive and negative predicted values for the final model were 80.4% (78.5–82.3% 95% CI) and 86.8% (85.7–87.9% 95% CI), respectively. Factors associated with stunting are birth weight (AOR = 0.79,p-value < 0.01), large infant size (AOR = 0.24,p-value < 0.05), mothers age (AOR = 0.98, p-value < 0.01), mothers height (AOR = 0.91, p-value < 0.01), fathers height (AOR = 0.95, p-value < 0.01), mothers lower education level(AOR = 1.50, p-value < 0.05), birth at health facilities (AOR = 0.83, p-value < 0.05), toilet standard(AOR = 0.86,p-value < 0.05), and waste disposal standard (AOR = 0.79 ,p-value < 0.01).

**Conclusions:**

A machine learning-based stunting prediction model effectively identifies a high-risk newborn population and enables early targeted interventions. Integration of the prediction with a prescriptive approach that considers causal pathways and evidence-based interventions is required for precise and sustainable prevention of stunting.

## Background

Stunting is a linear growth disorder caused by chronic malnutrition and chronic diseases, especially during the first 1,000 days of life. According to the World Health Organization (WHO), stunting is defined as height-for-age (HFA) below − 2 standard deviations (SD) from the median of the WHO child growth curve. Children with an HFA Z-score below − 2 SD are categorized as stunted, and below − 3 SD as severely stunted [1–4]. Child stunting is one of the most significant barriers to human development, worldwide. The WHO states that Indonesia is among the three countries with the highest prevalence of stunting in Southeast Asia after Timor Leste and India, with a stunting prevalence of 27.67% in 2019 [5, 6]. According to the 2023 Indonesian Health Survey, the prevalence of stunting in children under five years of age in Indonesia in 2023 was 21.5%, which was 0.1% lower than that in 2022 [7].

The causes of stunting are complex and multiple factors. The three main factors that contribute to growth retardation in children are low birth weight, an imbalanced diet, and medical history, especially during the first 1000 days of life (the golden period) [8]. Growth disorders that occur during the neonatal period include fetal growth disorders since the womb, which are caused by malnutrition and infections experienced by the mother. After a baby is born, this is exacerbated by other factors such as the introduction of foods other than breast milk for < 6 months, poor complementary feeding practices, poor access to clean water, poor environmental sanitation that results in diarrhea and gastrointestinal infections in children, recurrent infections, exposure to mycotoxins, arsenic, biomass fuels, poor infant stimulation and care, and maternal depression [9, 10]. Growth disorders result in linear growth retardation, increased morbidity, and death in children due to infections and delays in motor development and brain function [9, 11]. The long-term impacts of stunting include (1) the weak cognitive ability of children, (2) stunted psychomotor growth, (3) susceptibility to degenerative diseases, and (4) low-quality human resources in the future [10]. Therefore, to improve the management and control of the risk of stunting in children, a predictive model that can predict stunting early is needed. The early detection of risk factors and accurate diagnosis of stunting can play a key role in timely interventions to prevent and treat stunting.

Machine learning (ML) is a modern technology that falls under the umbrella of artificial intelligence (AI). Automated ML-based models that have been recently developed are in increasing demand because of their ability to predict the risk of malnutrition among children under the age of five in different countries [12]. Machine learning allows for the processing of large amounts of data through data mining to identify patterns and relationships that may be difficult to find using conventional statistical methods. Therefore, the purpose of this research is to produce a stunting prediction model algorithm using ML based on a data-mining framework that can predict the incidence of stunting in children in Indonesia since birth. This research is expected to contribute to the development of effective tools for the early identification of stunting risk so that health workers can implement prevention and intervention measures more quickly and accurately for a targeted newborn population.

## Methods

### Study design

This study is a quantitative research to develop the best prediction model utilizing machine learning algorithms as a core technology in artificial intelligence. The designed prediction model was intended for use in newborns to predict the incidence of stunting at the age of 2 years. The outcome variable in this study was stunting, which was calculated based on anthropometric indicators of height for children aged 24–35 months. The z-score of the children’s height according to age was calculated using the growth standards published by the WHO in 2006. The z-score of height for age is a metric used to assess linear growth retardation and cumulative growth deficits in children [13]. The identification of predictive factors for stunting in children refers to the results of a scoping review conducted from to 2012–2022 on the risk factors for stunting in children in Southeast Asia [14]. These predictive factors included child characteristics at birth, parental characteristics, household environmental health, and access to health services during pregnancy and childbirth.

### Data source

The study included data from all children aged two years in 1997, 2000, 2007, and 2014 from the Indonesia Family Life Survey (IFLS) [15]. The survey was conducted by the Research and Development (RAND) Corporation of the United States, in collaboration with the University of Indonesia and Gadjah Mada University. The IFLS data are available free of charge at https://www.rand.org/about.html [15, 16]. The variables taken from the IFLS data consisted of child characteristics including birth weight, sex, infant body size, and category of babies. Parental characteristics included age of the mother and father, height of the mother and father, parity, and gestational age. Access to health services included antenatal care status, number of antenatal care visits, consumption of fe tablets, and place of delivery. Socioeconomic factors included the educational level of the father and mother, employment status of the father and mother, smoking habits, source of drinking water, use of toilets according to standards, waste management, and waste management. There are 23 variables used as predictors of stunting. The selection of 23 predictor variables in this study was based on a previously published scoping review on stunting risk factors in Southeast Asia and the malnutrition framework issued by UNICEF, as well as the availability of IFLS data [14, 17]. Raw IFLS data were extracted using STATA MP-17.

### Statistical analysis

This study utilized the Cross-Industry Standard Process for Data Mining (CRISP-DM) framework in the data mining process including data preprocessing, modeling, and evaluating models [18]. Before performing modeling using machine learning, data exploration was first conducted using univariate analysis to see the frequency distribution and bivariate analysis to see the relationship between predictor factors and stunting cases, in addition to performing multivariate analysis to see the strength of the relationship. At the model creation stage, five machine learning algorithms were used to build the prediction model: random forest, naïve Bayes, neural network, decision tree, and K-nearest neighbor (kNN) using Orange Data Mining software (https://orangedatamining.com/docs/.) These models are part of the supervised learning paradigm and are known for their ability to be interpreted and widely used in the medical [19]. Five machine learning algorithms were compared (kNN, random forest, neural network, decision tree, and naïve Bayes) to empirically identify the best-performing model. Compared to classical statistical methods such as logistic regression, machine learning algorithms are more flexible and capable of modeling nonlinear relationships, handling high-dimensional data, and accommodating imbalanced datasets. This approach is increasingly relevant in the context of stunting data in Indonesia, which is multidimensional and exhibits high variability across populations.

Variable selection was conducted to identify and remove irrelevant or excessive features from the dataset to reduce the risk of overfitting, thereby improving system performance and ensuring fast and effective delivery of results [20]. At this stage, we used the information gain and Gini reduction methods, followed by the modeling stage. In this stage, researchers use supervised learning models with Naïve Bayes, artificial neural networks, kNN, decision trees, and random forests algorithm, with 10-fold cross-validation to identify the most accurate prediction model. In this study, the F1-score was chosen as the primary metric to evaluate the performance of the prediction model because the F1-score combines precision and recall into one harmonized value. Thus, it is expected to balance the model’s ability to detect positive cases correctly while avoiding false positive predictions [21]. As secondary metrics, the area under the (AUC) Receiver Operating Characteristic (AUC) curve, accuracy, sensitivity, and specificity of the candidate models were assessed to check the consistency of the results. The confusion matrix is used to present the actual and predicted values in tabular form and to calculate the positive and negative prediction values. As additional analysis, explanatory model was developed by multivariable logistic regression. For this purpose, descriptive statistical data analysis was performed using the chi-square test for categorical data and the t-test for numerical data. Further, multivariate analysis was performed using logistic regression with the stepwise backward method to produce the best explanatory model.

**Fig. 1:**
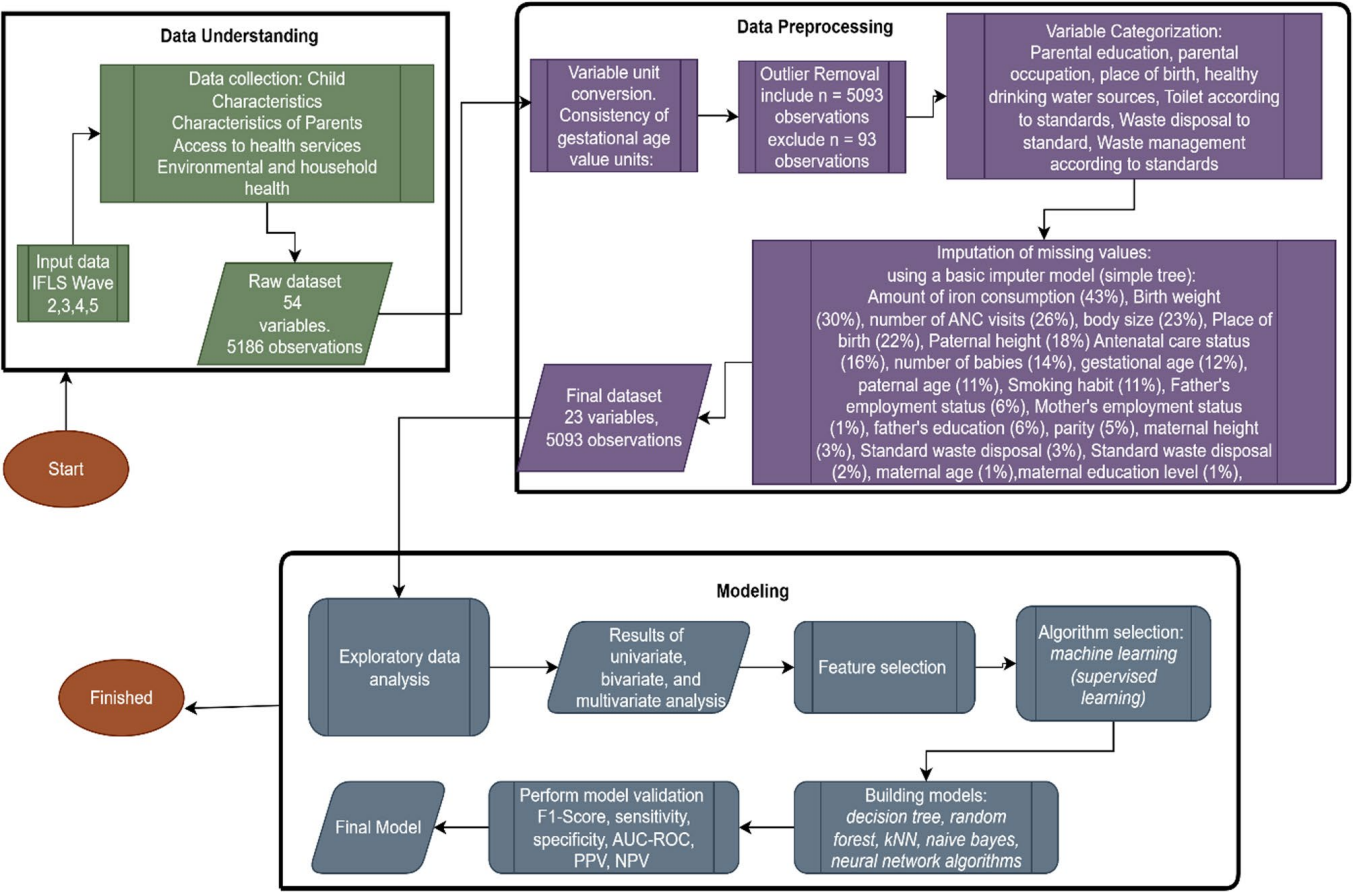
Stage of machine learning procedures

The overall analytical workflow of this study is summarized in Fig. 1, which illustrates each step taken from initial data understanding and preprocessing, through feature selection, model development, validation, and performance evaluation. This visual overview provides clarity on how the predictive model was built using supervised machine learning techniques and supports the transparency and reproducibility of the methodology.

Figure 1 shows stage of machine learning procedures, starting from the IFLS raw data consisting of 54 variables and 5186 observations, including stunting predictor variables and data identity. The overall analytical procedure is summarized in a structured flowchart (Fig. 1), which outlines the steps taken from data understanding and preprocessing to model building, validation, and evaluation. These steps follow the CRISP-DM framework and were designed to ensure a rigorous and transparent predictive modeling approach. The diagram includes the handling of missing data, feature selection strategies, algorithm comparison, and evaluation metrics to determine the best-performing model [22]. 

## Results

The IFLS dataset included 5093 children aged two years. The analysis of the dataset revealed that 35% (*n* = 1803) of the children presented with stunting, whereas the remaining 65% (*n* = 3290) did not. Before the modeling stage, researchers examined the data description used for modeling, as shown in Table 1 at the end of this document. Table 1 shows the initial association analysis of the predictor variables for stunting. Furthermore, a logistic regression analysis was conducted to produce explanatory model and to identify the factors associated with stunting. The variables included in this analysis were those with a p-value < 0.25 based on the results in Table 1.

**Table 1:** Univariable analysis of potential predictors for newborns to have stunting at 2-year-old

No	Predictors	*N* (%) or Mean (SD)			
		Normal *n* = 3290 (65%)	Stunting *n* = 1803 (35%)	All	*P* value
	Baby characteristics				
1	Birth weight (Kg), mean and SD	3.12(0.46)	3.07(0.46)	3.14(0.47)	< 0.001 ^a^
2	Gender of the child • Boys • Girls	1633 (49,6) 1657 (50.3)	920 (51.0) 883 (49.0)	2553(50.1) 2540(49.9)	0.361
3	Infant body size^c^ • Very large • Large • Normal • Small • Very Small	51 (1.5) 705 (21.4) 2198 (66.8) 333 (10.1) 3 (0.2)	5 (0.3) 325 (18.0) 1253 (69.5) 207 (11.5) 13 (0.7)	56 (1,1) 1030 (20.2) 3451 (67.8) 550 (10.6) 16 (0.3)	< 0.001 ^b^
4	Category of babies • Single • Twins	3185 (96.8) 105 (3.2)	1734 (96.2) 69 (3.8)	4919 (96.6) 174 (3.4)	0.191
	Parental characteristics				
5	Mother’s age (years), mean and SD	29.10 (5.8)	29.9 (6.1)	29.61 (5.94)	< 0.001 ^a^
6	Mother’s height (cm), mean and SD	151.8 (5.13)	149.5 (4.92)	151.28 (5.36)	< 0.001 ^a^
7	Father’s height (cm), mean and SD	163.45 (5.22)	161.75 (5.25)	162.74 (5.36)	< 0.001 ^a^
8	Father’s Age (year), mean and SD	34.21 (6.8)	33.97 (6.9)	34.16 (6.90)	0.294
9	Parity (n) mean and SD	1.5 (0.9)	1.5 (0.9)	1.51 (0.87)	0.760
10	Gestational age (weeks), mean and SD	36.37 (2.55)	36.38 (2.35)	36.34 (2.57)	0.985
	Access to health services				
11	Number of ANC visits (frequency), mean and SD	8.95 (2.9)	8.57 (2.8)	8.81 (2.89)	< 0.001 ^a^
12	Total iron consumption (total) mean and SD	33.4 (21.25)	33.85 (21.52)	33 (21.33)	0.559
13	Antenatal care status • Yes • No	3183 (96.7) 107 (3.3)	1715 (95.1) 88 (4.9)	4898 (96.2) 195 (3.8)	0.002 ^b^
14	Place of birth • health facilities • non-health facilities	2505 (74.1) 785 (23.9)	1181 (65.5) 622 (34.5)	3686 (72.4) 1407 (27.3)	< 0.001 ^b^
	Socioeconomic status				
15	Level of mother’s education • Low • Intermediate • High	1740 (52.9) 1047 (31.8) 503 (15.3)	1233 (68.4) 441 (24.5) 129 (7.1)	2973 (58.4) 1488 (29.2) 632 (12.4)	< 0.001 ^b^
16	Mother’s employment status • Working • Not working	1633 (49.6) 1657 (50.4)	850 (47.1) 953 (52.9)	2483 (48.7) 2610 (51.3)	0.115
17	Level of Father’s education • Low • Intermediate • High	1676 (50.9) 1112 (33.8) 502 (15.3)	1221 (67.7) 438 (24.3) 144 (8)	2897 (56.9) 1550 (30.4) 646 (12.7)	< 0.001 ^b^
18	Father’s employment status • Working • Not working	3248 (98.7) 42 (1.3)	1782 (98.8) 21 (1.2)	5030 (98.8) 63 (1.2)	0.061
	Environmental condition				
19	Smoking habits • Yes • No	2493 (75.8) 797 (24.2)	1421 (78.8) 382 (21.2)	3915 (76.8) 1218 (23.2)	0.016
20	Healthy drinking water sources • Yes • No	2478 (75.3) 812 (24.7)	1397 (77.5) 406 (22.5)	3875 (76) 1250 (24.1)	0.113
21	Toilet according to standards • Yes • No	2255 (68.5) 1035 (31.5)	969 (53.7) 834 (46.3)	3224 (63.3) 1869 (36.7)	< 0.001 ^b^
22	Waste disposal to standard • Yes • No	1938 (58.9) 1352 (41.1)	961 (53.3) 842 (46.7)	2899 (56.9) 2194 (43.1)	< 0.001 ^b^
23	Waste management according to standards • Yes • No	1081 (32.9) 2209 (67.1)	360 (20) 1443 (80)	1441 (28.3) 3652 (71.7)	< 0.001 ^b^

*SD* Standard deviation, *ANC* Antenatal Care

a: Statistical test comparing means

b: Statistical test using the chi-square test

^c^The infant’s body size was based on the parents’ perception during the interview.

Table 2 presents the results of the multivariate analysis of the risk factor model using logistic regression with the stepwise backward method. This final explanatory model showed that only nine predictor variables were associated with stunting with a p-value < 0.05, namely birth weight (kg), infant body size, mother’s age (years), mother’s height (cm), level of mother’s education, place of birth, father’s height (cm), toilet according to standards, and waste management according to standards.

**Table 2:** Multivariable analysis of potential predictors for newborns to have stunting at 2-year-old

No	Variable	Adjusted Odds Ratio (AOR)	95% CI (Lower–Upper)	*p*-value
	Baby Characteristic			
1	Birth weight (Kg)^b^	0.791	0.665–0.941	< 0.01*
2	Infant body size^b^ • Very large • Large • Normal • Small • Very Small	0.055 0.238 0.255 0.216 Ref	0.011–0.288 0.062–0.916 0.067–0.967 0.057–0.821 Ref	< 0.01* 0.037 0.045 0.025 Ref
3	Category of babies^b^ • Single • Twins	Ref 0.860	Ref 0.732–1.012	Ref 0.069
	Parental characteristics			
4	Mother’s age (years)^b^,	0.978	0.968–0.989	< 0.01*
5	Mother’s height (cm)^b^	0.911	0.900–0.923	< 0.01*
6	Father’s height (cm)^b^	0.952	0.940–0.964	< 0.01*
	Access to health services			
7	Antenatal care status^a^ • Yes • No	Ref 1.009	Ref 0.640–1.186	Ref 0.379
8	Number of ANC visits^a^	0.996	0.973–1.019	0.709
9	Place of birth^b^ • Healthcare facility • Non healthcare facility	0.830 Ref	0.720–0.957 Ref	0.010* Ref
	Socioeconomic status			
10	Level of mother’s education^b^ • Higher education • Secondary educational • Lower education	Ref 1.360 1.491	Ref 1.052–1.758 1.145–1.941	Ref 0.003* 0.019*
11	Mother’s employment status^a^	1.007	0.888–1.143	0.910
12	Level of Father’s education^b^ • Higher education • Secondary educational • Low education	Ref 0.924 1.257	Ref 0.973–1.187 0.973–1.623	Ref 0.538 0.080
13	Father’s employment status^a^	0.899	0.498–1.624	0.725
	Environmental condition			
14	Smoking habits^a^	1.070	0.922–1.242	0.374
15	Healthy drinking water sources^a^	1.029	0.888–1.192	0.707
16	Toilet according to standards^b^ • Yes • No	0.856 Ref	0.745–0.984 Ref	0.029* Ref
17	Waste disposal to standard^a^	1.091	0.957–1.244	0.193
18	Waste management according to standards^b^ • Yes • No	0.787 Ref	0.674–0.920 Ref	< 0.01* Ref

(^a^) Coefficient values obtained from full model

(^b^)Coefficient values obtained from final model

**Fig. 2:**
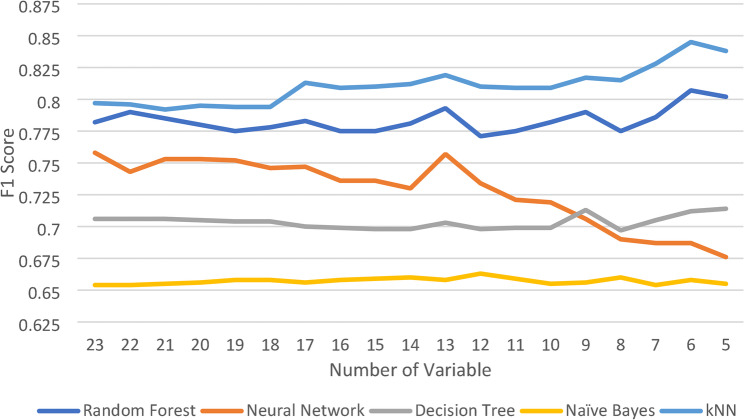
Performance of machine learning algorithms by the F1-score

Prediction modeling was conducted using a machine-learning approach with five predefined algorithms: random forest, neural network, decision tree, naïve Bayes, and k-nearest neighbor (kNN). The model used 23 variables that were selected incrementally to ensure an optimal model performance. Variable selection during the modeling process resulted in the most optimal model performance when the top six variables were used by the kNN algorithm, as determined by the results shown in Fig. 2.

Figure 2 shows the performance of the five machine learning algorithms used for stunting prediction modeling on the IFLS dataset. The figure shows that the kNN algorithm has the best overall performance compared with the other algorithms.

The six strongest predictor variables generated by the kNN algorithm are presented in Table 3.

**Table 3:** Variables of prediction model (Machine Learning) versus risk factor model (Logistic Regression)

No	Predictor factors	Information Gain value	Gini Decrease value	Risk Factor (*P* < 0.05)
1.	Mother’s height^a^	0.045	0.028	Yes
2.	Father’s height^a^	0.023	0.015	Yes
3.	Level of Father’s education^a^	0.020	0.013	No
4.	Level of Mother’s education^a^	0.019	0.012	Yes
5.	Toilet according to standards^a^	0.015	0.010	Yes
6.	Waste management according to standards^a^	0.014	0.009	Yes
7.	Place of birth	0.009	0.006	Yes
8.	Infant body size	0.006	0.004	Yes
9.	Mother’s age	0.005	0.003	Yes
10.	Number of ANC visits	0.005	0.003	No
11.	Birth weight	0.003	0.002	Yes
12.	Waste disposal to standard	0.002	0.001	No
13.	Father’s Age	0.002	0.001	No
14.	Parity	0.001	0.001	No
15.	Antenatal care status	0.001	0.001	No
16.	Gestational age	0.001	0.001	No
17.	Smoking habits	0.001	0.001	No
18.	Total iron consumption	0.001	0.000	No
19.	Category of babies	0.000	0.000	No
20.	Healthy drinking water sources	0.000	0.000	No
21.	Mother’s employment status	0.000	0.000	No
22.	Gender of the child	0.000	0.000	No
23.	Father’s employment status	0.000	0.000	No

*ANC* Antenatal Care

^a^Predictors in bold are included in the final prediction model

A thorough examination of the available datasets revealed six factors identified as the best predictors of stunting in two-year-old children. These factors include maternal and paternal height, educational attainment of both parents, ownership of latrines adhering to established standards, and management of waste according to specified criteria. An examination of the data in Table 3 reveals the presence of these features. The selection of the six factors was based on the information gain and Gini decrease values of each predictor variable. Information gain is a feature selection method that ranks variables that are considered the most influential in stunting prediction. This assessment method considers the interactions between variables and reflects their importance in building a prediction model [23]. Table 3 also shows the risk factors for stunting based on the results of logistic regression. Statistically significant risk factors are not necessarily included in the best prediction model.

As illustrated in Table 4, the final modeling results indicate the highest prediction value. Among the five algorithms employed, the k-NN algorithm consistently demonstrated the optimal performance metric. This assertion is substantiated by the F1-score value of 0.845, which serves as a reliable metric for evaluating the performance of machine-learning models.

**Table 4:** Machine learning algorithm performance for predicting stunting in 2-year-old children

Assessment metrics	Naïve Bayes	Neural Network	Decision Tree	Random Forest	kNN
F1-score	0.658	0.687	0.712	0.805	0.845
AUC-ROC	0.682	0.725	0.755	0.862	0.914
Sensitivity	0.661	0.699	0.714	0.811	0.847
Accuracy	0.661	0.699	0.714	0.811	0.847

*AUC-ROC Area under the curve–receiver operating characteristic

*kNN K-nearest neighbor

Table 5 presents a confusion matrix assessment of the kNN algorithm. Based on the results of the k-nearest neighbors (kNN) algorithm evaluation of data for 2-year-old children, a positive predictive value (PPV) of 80.38% and a negative predictive value (NPV) of 86.78% were obtained.

**Table 5:** Calculation of confusion matrix values ​​in the kNN algorithm for 2-year-old children

		Predicted		
		Stunting	Normal	N
Observed	Stunting	TP = 1352	FN = 451	1803
	Normal	FP = 330	TN = 2960	3290
	N	1682	3411	5093

*TP True positive, *FP* False positive, *FN* False negative, *TN* True negative

## Discussion

This study successfully developed a first machine learning model to predict newborns in Indonesian population who have the potential to experience stunting at the age of two years based on IFLS data with sample size of 5093 children subjects. Basically, our model is a statistical model built by machine learning algorithm to predict or estimate the risk or probability of a future event (outcome) occurring in an individual or group based on the input data (predictors or independent variables) [24, 25] with F1 score as an accuracy metric of 84.5%. This stunting prediction model accuracy is not far relatively with other study in Bangladesh and Ghana, which showed accuracy of 82% and 98%, with sample size of 8,759 and 8,564 children subject respectively. Given the final model, we will expect to have approximately 80.4% positive predictive value (PPV) and 86.8% negative predictive value (NPV). For example, if there were two hypothetical newborn subjects with different characteristics as showed in Table 6, the prediction model will produce the probability of 0.21 and 0.9 and predict that the first subject to have not stunting and second subject to have stunting at 2 years old, assuming that no special intervention will be given. Translating the PPV and and NPV, for every 10 children predicted to have stunting, 8 children will have it and potentially to be prevented.

**Table 6:** Prediction on Two-Hypothetical subjects by the final prediction model

Actual	Stunting Prediction	Mother’s height (cm)	Father’s height (cm)	Level of mother’s education	Level of Father’s education	Toilet according to standards	Waste management according to standards	Stunting Probability	Normal Probability
No	No	164	166	Lower	Secondary	Yes	Yes	0.21	0.78
Yes	Yes	145	161.5	Lower	Lower	No	No	0.93	0.06

Table 6 presents a practical demonstration of how the final prediction model can be applied to individual cases. Two hypothetical newborns with contrasting characteristics are shown to have markedly different predicted probabilities of stunting at age two (0.21 vs. 0.93). This table illustrates the clinical and public health utility of the model in identifying high-risk newborns and enabling early targeted interventions. It highlights how small differences in key predictor variables can significantly affect the predicted outcome, emphasizing the model’s potential application in individual risk assessment and personalized prevention strategies.

Although our final prediction model seems promising in identifying higher risk newborn for stunting and suggesting early targeted prevention, there are challenges and implications on what will be required for the prediction model to make a true impact of stunting prevention in the population. This study showed that the machine learning process needs adequate data input to provide adequate statistical power and to produce acceptable prediction accuracy. In Indonesia context, where under-five children compose about 5% of population, data from at least a total 100,000 population size or 25,000 family will be needed to collect about 5,000 children’s observation and to produce such model. In our study, we used family survey project (IFLS) data that had been collected between 1997 and 2014, with high volume training and standardization effort to ensure the data quality and in fact, this usage of high quality ad hoc-survey data in building prediction model is common [12, 26]. Therefore, to establish real-time valid and reliable prediction, the quality and completeness of regular-intersectoral-data collection workflow within existing health care system should be in place beforehand [27].

In addition to data quality and completeness as part of the model internal validity, the population in which the data were collected from will also greatly determine the generalization of the machine learning model to other population or the external validity [28]. This is a universal challenge for machine learning model that now very widely used in artificial intelligence technology created to support health care decision making. For example, in term of space, the children IFLS data used in this study came from 13 out of 38 provinces in Indonesia and therefore it would be questionable if this model could be generalized to other provinces since the prediction model might have lower accuracy if applied to children from outside those 13 provinces. The IFLS data used in this study were collected between 1997 and 2014. Thus, all data used are historical and publicly available. No current or future data were involved in this study. We acknowledge, however, that data collected more than a decade ago may not fully reflect today’s population characteristics. Therefore, to improve the future applicability of such models, updated and routinely collected local data are recommended. If those 5000 children subjects from only 1 province, the prediction model will have greater external validity for application to that province. In this context, this what so called local prediction model building arguably will provide better model fit and improve its external validity when applied locally today.

As we mentioned earlier that this prediction model will have impact if only it follows by effective prevention program targeted to the subjects who are being predicted to have stunting. In this context, knowledge on cause-and-effect relationship between modifiable factors and stunting would provide the fundamental evidence for implementing reasonable preventive program. For this purpose, we build the explanatory models based on the same data and able to identify 9 associated factors. This explanatory model basically a population or global or marginal model which identify general factors associated with stunting, but not particular factor to individual. Two children may have similar stunting prediction, yet they have different factors associated with it such as mother’s age and education level for example. Although mother’s age and education background are considered non-modifiable factor, it might influence the baseline of mother knowledge, attitude, and practice that eventually would provide the context of the stunting prevention approach such as education or counselling program. For modifiable factors such as birth weight, infant body size, place of birth, toilet according to standards, and waste management according to standards, the conditions that relevant to a children will certainly have implication on the possible specific stunting pathway and its personalized prevention. Hence, unlike the prediction model with main purpose is to generate forecasts and that identify who should be intervened, the explanatory model designed to test hypotheses about the cause-and-effect relationships between explanatory and outcome variable. The goal of an explanatory model is to understand the etiological mechanisms of stunting, and therefore will guide more on the decision of what and how to be intervened [29, 30].

The combination of predictive and explanatory models will lead to a more comprehensive understanding of the dynamics of stunting, as well as open up space for the formulation of more rigorous prescriptive models. Prescriptive models play an important role as a basis for designing prevention interventions that are personalized and contextualized [31]. Stunting is the result of a complex and multifactorial process, which is not merely influenced by nutritional deficiencies, but also by maternal health conditions, socioeconomic status, recurrent infections, feeding practices, and environmental factors such as clean water and sanitation [17, 32]. Therefore, a general-nonspecific or one size fit for all approach typically unable to answer the specific needs of each individual. By knowing the children who have the potential to experience stunting and the underlying factors, the policies and intervention programs run by the government to reduce the prevalence of stunting in the golden period will be more focused, targeted, and efficient. So that interventions are no longer generic, but based on data and real needs, and this will have a more optimal impact in reducing the prevalence of stunting.

This study has shown the potential merit of prediction and prescriptive model with the support of machine learning as a core technology of artificial intelligent in supporting health care decision making for stunting prevention. Unfortunately, the use of IFLS data limits the applicability of the stunting prediction model from this study into Indonesia current health system. Nevertheless, here we would like to provide a reflective discourse for the future roadmap on the mission of stunting prevention by the artificial intelligence technology based on the discussion in previous section. The advantage of a data-driven approach, such as our stunting prediction model, lies in its ability to be continuously updated as new data becomes available, thus enabling adaptive learning and increasingly accurate predictions over time. Therefore, stunting prediction models require reliable algorithms and a robust data ecosystem beyond electronic medical record. This includes health information system that supports data integration across sectors, interoperability standards, and human resources with high data literacy [27, 33]. We believe that it is imperative to build a population-based health registry which contain longitudinal data in a well-defined area or local health system. Initially, this effort should be guided by the assessment of the big data ecosystem readiness in the local health system with the emphasize on the human resources capacity, Investment in human resource capacity building through technical training, strengthening data governance, and establishing cross-sectoral work units that focus on monitoring data quality and utilization is crucial [34, 35]. Without strengthening in these technical and institutional aspects, the study of prediction and prescription models in stunting prevention risks becoming just a technological initiative that has no real impact.

## Conclusion

This study shows that machine-learning-based stunting prediction models can identify high-risk children from birth, enabling targeted and efficient interventions. However, the effectiveness of predictive approaches depends on their integration with prescriptive models that understand the causal pathways and recommend evidence-based interventions. Strengthening data infrastructure, system interoperability, and human resource capacity are important prerequisites for an adaptive, precise, and sustainable stunting prevention program.

## Data Availability

The data presented in this study are available in the manuscript. The dataset used in this study is publicly available and can be accessed through the RAND Corporation website at https://www.rand.org/well-being/social-and-behavioral-policy/data/FLS/IFLS/download.html The authors used data from IFLS waves 2 to 5, which are open-access and do not contain any personal identifiers.

## Abbreviations

AUC: Area Under the Curve
ANC: Antenatal Care
CRISP-DM: Cross-Industry Standard Process for Data Mining
CI: Confident Interval
CM: Centimeter
FN: False negative
FP: False positive
IFLS: Indonesian Family Life Survey
Kg: Kilogram
kNN: K-Nearest Neighbor
ML: Machine Learning
NPV: Negative predictive value
PPV: Positive predictive value
RF: Random Forest
ROC: Receiver Operating Characteristic
TP: True positive
TN: True negative
WHO: World Health Organization
